# Impact of age on indication for chemotherapy in early breast cancer patients: results from 104 German institutions from 2008 to 2017

**DOI:** 10.1007/s00404-022-06902-9

**Published:** 2023-01-05

**Authors:** Ann Sophie Hoffmann, André Hennigs, Manuel Feisst, Mareike Moderow, Sabine Heublein, Thomas Maximilian Deutsch, Riku Togawa, Benedikt Schäfgen, Markus Wallwiener, Michael Golatta, Jörg Heil, Fabian Riedel

**Affiliations:** 1 Department of Gynecology and Obstetrics, Heidelberg University Hospital, Im Neuenheimer Feld 440, 69120 Heidelberg, Germany; 2 Institute of Medical Biometry, Heidelberg University Hospital, Heidelberg, Germany; 3 West German Breast Center (WBC) GmbH, Düsseldorf, Germany; 4 National Center for Tumor Diseases, Heidelberg, Germany; 5 Heidelberg Breast Center at the St. Elisabeth Clinic, Heidelberg, Germany

**Keywords:** Early breast cancer, Pathological complete response, Neoadjuvant chemotherapy, Age, Elderly patients

## Abstract

**Purpose:**

Today, the decision to treat patients with chemotherapy for early breast cancer (EBC) is made based on the patient’s individual risk stratification and tumor biology. In cases with chemotherapy indication, the neoadjuvant application (NACT) is the preferred option in comparison with primary surgery and adjuvant chemotherapy (ACT). Age remains a relevant factor in the decision-making process. The aim of the present study was to illustrate the impact of age on the use of systemic therapy in clinical routine.

**Methods:**

The study separately analyzed chemotherapy use among six age cohorts of EBC patients who had been treated at 104 German breast units between January 2008 and December 2017.

**Results:**

In total, 124,084 patients were included, 46,279 (37.3%) of whom had received chemotherapy. For 44,765 of these cases, detailed information on treatment was available. Within this cohort, chemotherapy was administered as NACT to 14,783 patients (33.0%) and as ACT to 29,982 (67.0%) patients. Due to the higher prevalence of unfavorable tumor subtypes, younger patients had a higher rate of chemotherapy (≤ 29y: 74.2%; 30–39y: 71.3%) and a higher proportion of NACT administration ( ≤ 29y: 66.9%; 30–39y: 56.0%) in comparison with elderly patients, who had lower rates for overall chemotherapy (60–69y: 37.5%; ≥ 70y: 17.6%) and NACT (60–69y: 25.5%; ≥ 70y: 22.8%). Pathologic complete response was higher in younger than in older patients (≤ 29y: 30.4% vs. ≥ 70y: 16.7%), especially for HER2− subtypes.

**Conclusion:**

The data from the nationwide German cohort reveal relevant age-dependent discrepancies concerning the use of chemotherapy for EBC.

## What does this study add to the clinical work

**Table:** 

Data from a nationwide German cohort reveal relevant discrepancies concerning the indication for and patterns of chemotherapy use for early breast cancer depending on age. Younger patients (< 40 years) more often receive chemotherapy both in general and in a neo adjuvant therapy setting. These younger patients also have higher rates of pathologic complete remission in comparison with elderly patients, especially for HER− subtypes.	

## Introduction

Mortality in early breast cancer (EBC) has declined over the past decade in most developed countries — such as Germany [1] — due to new developments in screening, diagnostics, surgery, radiotherapy, and systemic therapy, due to structural improvements (e.g., multidisciplinarity, specialized breast cancer units), and due to quality improvement measures, such as evidence-based guidelines [2]. A better molecular understanding of EBC [3] suggests that systemic therapy for EBC should be tailored according to individual risk factors and intrinsic subtypes [4].

In the past decade, this process has led to a substantial decline in overall chemotherapy use in EBC due to the availability of more individualized treatment decisions. However, the expanding application of neoadjuvant chemotherapy (NACT) (in comparison with adjuvant chemotherapy; ACT) has caused more patients to have a pathological complete response (pCR), which can be regarded as a surrogate for better outcomes (in comparison with non-pCR). These developments have been demonstrated for Germany in previous single-center [5] and multicenter [6] analyses.

Although the indication for chemotherapy in EBC is mainly driven by tumor biology, age remains a relevant factor in routine decision-making. Very young and old age are particularly important factors that might impact treatment decisions: When it comes to defining which EBC patients should be considered young, the limit can be set at 40 years or younger, in keeping with recent ESMO guidelines [7]. This group of patients represents around 5% of all EBC patients [8], albeit with a rising incidence [9]. When it comes to elderly EBC patients, defining a threshold for specific therapy management is more difficult because numerical age is influenced by individual performance and frailty, with a threshold of  ≥ 70 years often being used to define the group [10]. Elderly patients with comorbidities are particular often underrepresented or excluded from clinical trials [11].

No nationwide tumor registration exists in Germany, and details about the indication for chemotherapy in the cohorts of both very young and elderly EBC patients, therefore, remain unclear, as does the impact of age on treatment patterns and outcomes for EBC within the German healthcare system. The aim of the present study was, thus, to illustrate both the impact of age on systemic treatment patterns for EBC and the respective outcomes of these treatment patterns among patients by using data from a large patient cohort derived from the clinical routine. For this purpose, we present data from 124,084 patients who were treated at 104 German institutions between 2008 and 2017.

## Methods

### Database

The present study uses data from the West German Breast Center GmbH (WBC), Düsseldorf, Germany [12]. Participating hospitals and breast cancer units (BCUs) contribute clinical, surgical, and pathological data on patients with EBC to the database, and the collaborating institutions collect the data prospectively. Thus, the present study represents a post hoc analysis of a prospectively collected database. The dataset does not include follow-up information on oncological outcomes.

For the analysis, anonymized data from all female patients with invasive EBC who had been treated between 1 January 2008 and 31 December 2017 were extracted from the database. The final dataset comprised 124,084 patients. EBC was defined as primary (non-metastasized) breast cancer that was being treated in curative intention. All patients had undergone breast surgery. The division into adjuvant and neoadjuvant chemotherapy was determined based on the date of surgery. Patients who had received both neoadjuvant and adjuvant (i.e., post-neoadjuvant) chemotherapy were subsumed as neoadjuvant (because neoadjuvant therapy was the primary therapy in these cases).

The study was approved by the Ethics Committee of Heidelberg University and was conducted in accordance with the Declaration of Helsinki. The study was deemed to be without risk because it included only analyses of routinely collected anonymized data. Consequently, the Ethics Committee did not request approval for consent for this designated analysis. Informed consent to analyze the anonymized data was obtained from all individual participants before data acquisition as part of the benchmarking process.

### Categorization of age groups

All patients were categorized into one of six different age groups, which were defined by the date of the patient’s (first) histopathologic diagnosis of EBC: Group 1: ≤ 29 years; Group 2: 30–39 years; Group 3: 40–49 years; Group 4: 50–59 years; Group 5: 60–69 years; and Group 6: ≥ 70 years.

### Definitions of tumor histology, stages, and subtypes

Tumor histology was defined according to the World Health Organization criteria [14], and post-operative pathological staging was performed in line with the recent TNM classification [15]. Response to NACT was determined using the post-operative specimens along international standards, and pCR after NACT was defined as ypT0 ypN0 – that is, as the absence of invasive cancer in breast and axillary lymph nodes. The expression of the immunohistochemical (IHC) parameters of estrogen receptor (ER), progesterone receptor (PR), human epidermal growth factor receptor 2 (HER2), and Ki-67 was assessed using formalin-fixed, paraffin-embedded tumor tissue according to international standards. For patients who were receiving NACT, IHC was based on the pre-treatment biopsy (if available); whereas for patients with ACT, IHC was based on the final post-operative pathological sample. The detailed criteria for positivity of the hormone receptors (HR) — that is, ER and PR — and of the HER2 status has been described previously [16]. HR was defined as negative if both ER/PR were negative and as positive if either ER or PR (or both) were positive. We then defined four subtypes: (1) HR+ and HER2− , (2) HR+ and HER2+ , (3) HR− and HER2+ , and (4) HR− and HER2−  (i.e., “triple negative”; TN).

### Statistical analysis

Annual percentages of chemotherapy use were calculated and presented in a longitudinal time-trend analysis for the period from 2008 to 2017 (in %) for the entire cohort. pCR rates were calculated from the subgroup of patients who had received NACT. All cases were assigned to a year (2008–2017) according to the date of the first histopathological documentation. Multivariable logistic regression modeling was used to identify factors associated with the achievement of pathological complete remission after NACT had been applied. Due to the extensive sample size of the register database, *p* values of < 0.05 were considered statistically significant in a descriptive sense. Missing data were not imputed. Data were analyzed descriptively using both SPSS software version 25 (IBM; Armonk, NY; USA) and R version 3.5.0.

## Results

### Patient and tumor characteristics

**Fig. 1:**
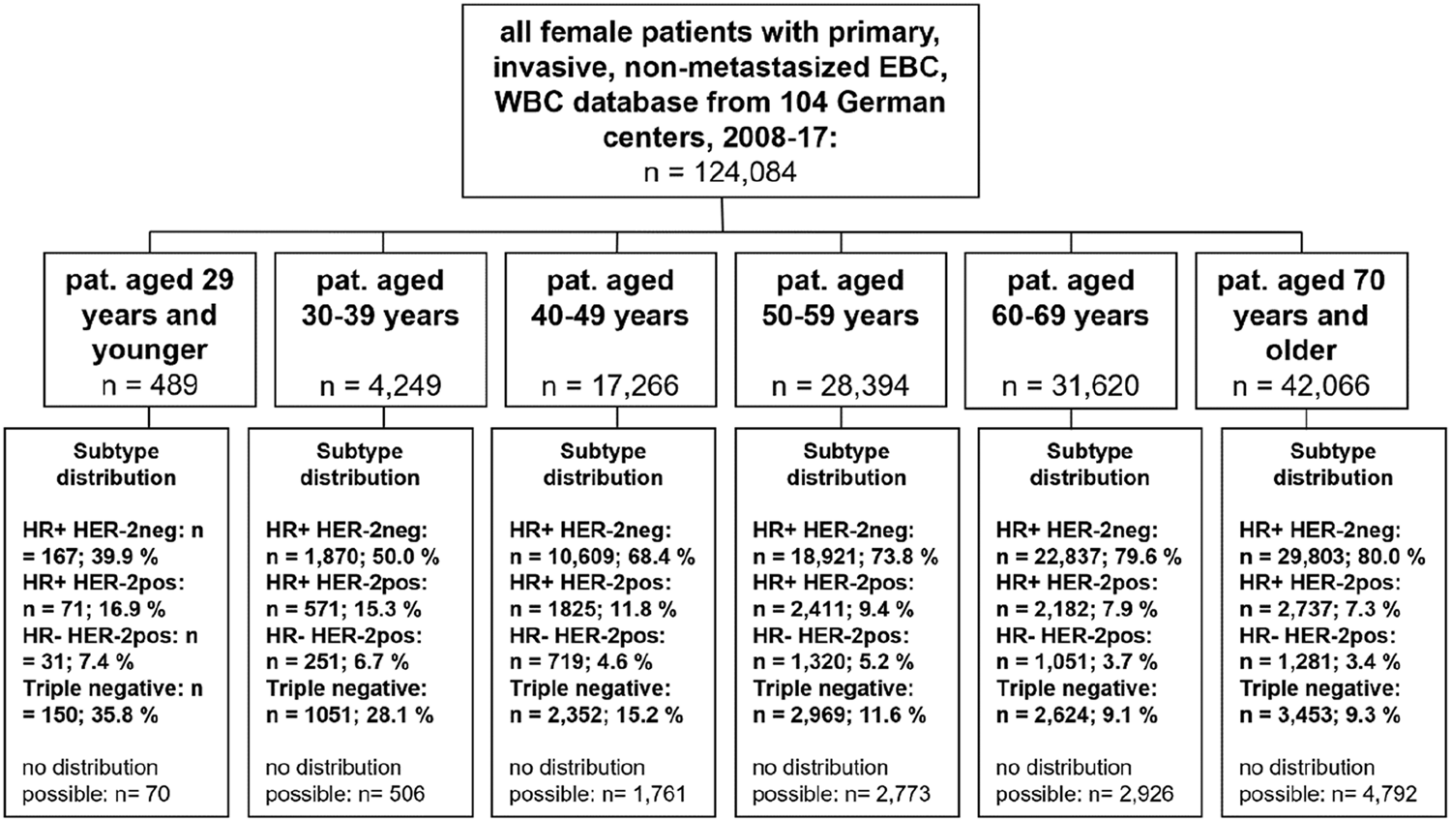
Patient cohorts

In total, 104 institutions provided a final dataset of 124,084 patients with EBC, 82.3% (*n* = 102,080) of whom were 50 years or older upon first diagnosis. Figure 1 presents the distribution among the six age cohorts. Menopause status relates to age, with nearly all women below age 30 being registered as pre-menopausal (95.0%) and nearly all women aged 70 and older being registered as post-menopausal (98.5%). Overall, most patients presented with tumors of stage T1/T2, with no relevant differences between the age groups. Higher tumor stages—classified as T3/T4—were most prevalent in the oldest age group and affected 6.0% and 5.4%, respectively, of the women in this group. In all other age groups, T3 and T4 tumors were less prevalent and affected between 2.6% (patients aged 60–69y) and 4.3% (patients aged 30–39y) as well as between 0.7% (patients aged 30–39y) and 1.4% (patients aged 60–69y), respectively, of the women in these groups. Overall, there was no relevant difference concerning nodal status, with most patients being nodal negative in all age groups. Regarding grading, most tumors in patients under 30 years old were graded as G3 (57.2%); whereas, most tumors in patients aged 40 and older were graded as G2 (55.8, 57.2, 61.6, and 63.1%, respectively). In relation to tumor subtype, the youngest age group displayed a rather unfavorable subtype distribution, with only 39.9% of patients presenting with the subtype HR+ HER2–, 16.9% presenting with the subtype HR+ HER2+ , 7.4% presenting with the subtype HR– HER2+ , and 35.8% presenting with the subtype HR– HER2–. In contrast, in the oldest age group, most patients presented with the subtype HR+ HER2– (80.0%), with other subtypes being relatively rare (HR+ HER2+ : 7.3%; HR– HER2+ : 3.4%; HR– HER2–: 9.3%). The younger the patient group was, the more often its members were being treated at a university hospital, with almost one-third of patients aged 29 or younger (28.6%) and only 8.5% of patients aged 70 or older being treated there. The Karnofsky Performance Status Scale indicates that functional impairment was more present in the older patient groups, with 71.8% and 20.1% of patients under 30 achieving a score of 100 or 90, respectively, while only 33.5% and 34.5%, respectively, of patients aged 70 years and older achieved the same score (Table 1).

**Table 1:** Patient and tumor characteristics for all cases of early breast cancer, divided into six age groups (Group 1: ≤ 29 y; Group 2: 30–39 y; Group 3: 40–49 y; Group 4: 50–59 y; Group 5: 60–69 y; Group 6: ≥ 70 y; total n = 124,084)

Patient characteristics (*n* = 124,084)												
	≤ 29 y (*n* = 489)		30–39 y (*n* = 4249)		40–49 y (*n* = 17,266)		50–59 y (*n* = 28,394)		60–69 y (*n* = 31,620)		≥ 70 y (*n* = 42,066)	
	Number	%	Number	%	Number	%	Number	%	Number	%	Number	%
Menopause status												
Pre	453	95.0	4008	95.6	14,020	82.2	5410	19.4	201	0.6	141	0.3
Peri	13	2.7	87	2.1	1405	8.2	3532	12.6	348	1.1	473	1.1
Post	11	2.3	99	2.4	1621	9.5	18,999	68.0	30,607	98.2	40,735	98.5
Total	477	100	4194	100	17,046	100	27,941	100	31,156	100	41,349	100
Missing	12		55		220		453		464		717	
pT stadium (cases without neoadjuvant chemotherapy)												
pT1	91	60.7	1091	57.8	6056	58.6	13,029	66.7	15,932	66.6	13,503	42.0
pT1mic	1	0.7	6	0.3	15	0.1	64	0.3	55	0.2	47	0.1
pT2	50	33.3	683	36.2	3723	36.0	5615	28.8	6865	28.7	14,644	45.5
pT3	6	4.0	92	4.9	431	4.2	618	3.2	698	2.9	2079	6.5
pT4	2	1.3	16	0.8	103	1.0	204	1.0	363	1.5	1878	5.8
Total	150	100	1888	100	10,328	100	19,530	100	2,3913	100	32,151	100
Missing	102		692		2901		4514		4789		8343	
ypT stadium (cases with neoadjuvant chemotherapy)												
ypT0	84	39.1	539	36.2	1120	30.7	1200	30.8	770	29.6	329	24.4
ypTis	25	11.6	159	10.7	369	10.1	342	8.8	210	8.1	102	7.6
ypT1	64	29.8	458	30.7	1237	33.9	1291	33.1	882	33.9	422	31.3
ypT1mic	1	0.5	14	0.9	15	0.4	33	0.8	20	0.8	14	1.0
ypT2	29	13.5	241	16.2	705	19.3	800	20.5	509	19.6	338	25.1
ypT3	11	5.1	68	4.6	165	4.5	135	3.5	121	4.6	72	5.3
ypT4	1	0.5	11	0.7	42	1.1	98	2.5	91	3.5	72	5.3
Total	215	100	1490	100	3653	100	3899	100	2603	100	1349	100
Missing	22		179		384		451		315		223	
(y)pN stadium												
(y)pN0	297	70.9	2420	64.7	9967	64.3	17,874	69.5	20,802	73.0	21,731	64.1
(y)pN1	80	19.1	837	22.4	3507	22.6	4854	18.9	4702	16.5	6818	20.1
(y)pN1mi	15	3.6	113	3.0	517	3.3	815	3.2	717	2.5	799	2.4
(y)pN2	20	4.8	267	7.1	1,019	6.6	1426	5.5	1,419	5.0	2717	8.0
(y)pN3	7	1.7	106	2.8	488	3.1	738	2.9	870	3.1	1,832	5.4
Total	419	100	3743	100	15,498	100	25,707	100	28,510	100	33,897	100
Missing	70		506		1768		2687		3110		8169	
Grading												
G1	12	3.3	209	6.1	1811	12.2	4230	16.8	4770	16.6	4129	11.6
G2	146	39.6	1532	45.0	8295	55.8	14,430	57.2	17,654	61.6	22,477	63.1
G3	211	57.2	1667	48.9	4764	32.0	6588	26.1	6229	21.7	8988	25.3
Total	369	100	3408	100	14,870	100	25,248	100	28,653	100	35,594	100
Missing	120		841		2396		3146		2967		6472	
Estrogen-receptor status												
Positive	230	53.9	2385	62.5	12,379	78.2	21,370	81.7	25,158	86.1	32,975	86.3
Negative	197	46.1	1432	37.5	3452	21.8	4788	18.3	4059	13.9	5251	13.7
Total	427	100	3817	100	15,831	100	26,158	100	29,217	100	38,226	100
Missing	62		432		1435		2236		2403		3840	
Progesterone-receptor status												
Positive	202	47.3	2171	56.9	11,625	73.4	18,769	71.8	21,644	74.1	28,200	73.8
Negative	225	52.7	1646	43.1	4204	26.6	7380	28.2	7567	25.9	10,016	26.2
Total	427	100	3817	100	15,829	100	26,149	100	29,211	100	38,216	100
Missing	62		432		1437		2245		2409		3850	
HER2-receptor status												
Positive	104	24.8	825	22.0	2539	16.3	3687	14.4	3,205	11.1	3993	10.7
Negative	316	75.2	2932	78.0	12,997	83.7	21,997	85.6	25,559	88.9	33,342	89.3
Total	420	100	3757	10	15,536	100	25,684	100	28,764	10	37,335	100
Missing	69		492		1730		2710		2856		4731	
Subtype distribution												
HR+ HER2−	167	39.9	1870	50.0	10,609	68.4	18,921	73.8	22,837	79.6	29,803	80.0
HR+ HER2+	71	16.9	571	15.3	1825	11.8	2411	9.4	2182	7.6	2737	7.3
HR− HER2+	31	7.4	251	6.7	719	4.6	1320	5.2	1051	3.7	1281	3.4
HR− HER2−	150	35.8	1051	28.1	2352	15.2	2969	11.6	2624	9.1	3453	9.3
Total	419	100	3743	100	15,505	100	25,621	100	28,694	100	37,274	100
Missing	70		506		1761		2773		2926		4792	
Hospital-type distribution												
University	140	28.6	929	21.9	2622	15.2	3574	12.6	3557	11.2	3575	8.5
Teaching hospital	249	50.9	2304	54.2	10,260	59.4	17849	62.9	20,244	64.0	27,430	65.2
Other	100	20.4	1016	23.9	4384	25.4	6971	24.6	7819	24.7	11,061	26.3
Total	489	10	4249	100	17,266	100	28,394	100	31,620	100	42,066	100
Chemotherapy												
Yes	363	74.2	3031	71.3	10,261	59.4	13,363	47.1	11,848	37.5	7408	17.6
No	126	25.8	1218	28.7	7005	40.6	15,031	52.9	19,772	62.5	34,658	82.4
Total	489	100	4249	100	17,266	100	28,394	100	31,620	100	42,066	10
Chemotherapy with complete information available												
NACT	237	66.9	1669	56.0	4037	40.1	4350	33.4	2918	25.5	1572	22.8
ACT	117	33.1	1309	44.0	6019	59.9	8679	66.6	8540	74.5	5318	77.2
Total	354	100	2978	100	10,056	100	13,029	100	11,458	100	6890	100
Karnofsky performance status scale												
0	0	0.0	0	0.0	0	0.0	0	0.0	0	0.0	0	0.0
10	0	0.0	0	0.0	0	0.0	0	0.0	0	0.0	0	0.0
20	0	0.0	0	0.0	1	0.0	3	0.0	3	0.0	15	0.0
30	0	0.0	0	0.0	3	0.0	8	0.0	13	0.1	24	0.1
40	0	0.0	1	0.0	6	0.0	25	0.1	36	0.1	167	0.6
50	2	0.6	5	0.2	27	0.2	40	0.2	88	0.4	672	2.2
60	2	0.6	11	0.4	37	0.3	77	0.3	155	0.6	1112	3.7
70	1	0.3	11	0.4	91	0.7	240	1.1	496	2.0	2136	7.1
80	22	6.6	230	7.4	850	6.4	1756	7.9	2544	10.2	5538	18.3
90	67	20.1	711	23.0	3312	24.9	6279	28.4	7943	32.0	10,433	34.5
100	239	71.8	2128	68.7	8995	67.5	13,709	61.9	13,538	54.5	10,111	33.5
Total	333	100	3097	100	13,324	100	22,138	100	24,820	100	30,212	100
Missing	156		1152		3944		6257		6804		11,858	

### Chemotherapy use

**Fig. 2:**
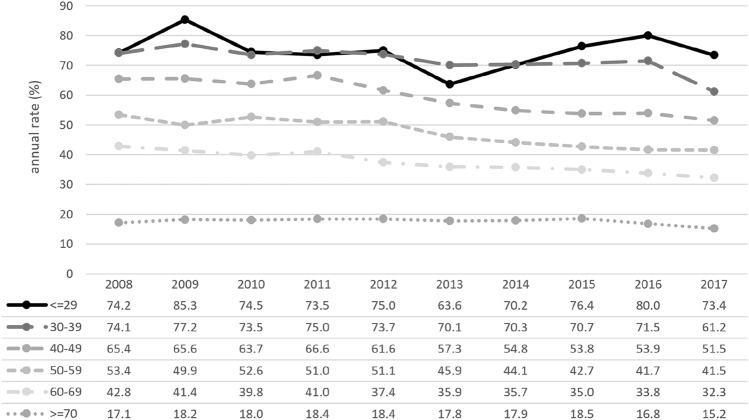
Overall portion of patients receiving chemotherapy (CHT), divided into six age groups (Group 1: ≤ 29 years; Group 2: 30–39 years; Group 3: 40–49 years; Group 4: 50–59 years; Group 5: 60–69 years; Group 6: ≥ 70 years; total *n* = 124,084)

**Fig. 3:**
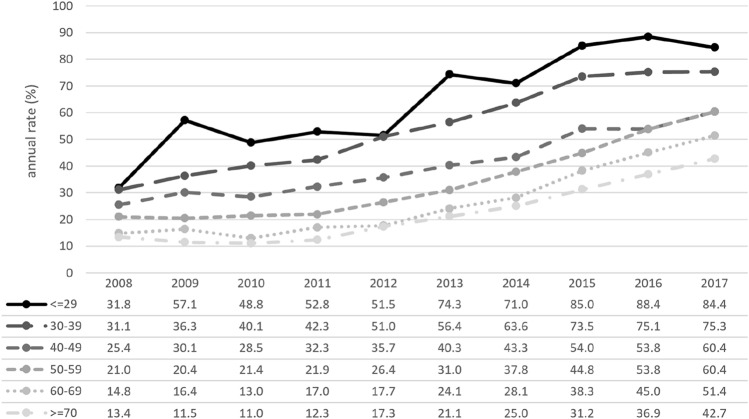
Relative portion of neoadjuvant chemotherapy (NACT) use (among all patients on chemotherapy), divided into six age groups (Group 1: ≤ 29 years; Group 2: 30–39 years; Group 3: 40–49 years; Group 4: 50–59 years; Group 5: 60–69 years; Group 6: ≥ 70 years; total *n* = 44,765; missing *n *= 1509)

In total, 46,274 (37.3%) patients had received chemotherapy, 44,765 of whom had complete information available and 1,509 (3.3%) of whom had missing data on treatment. Of the patients with complete information, 29,982 (67.0%) had received chemotherapy as ACT, and 14,783 (33.0%) had received chemotherapy as NACT. In total, 1,367 patients had received both neoadjuvant and adjuvant chemotherapy. Younger patients had received chemotherapy more often both overall (≤ 29y: 74.2%; 30–39y: 71.3%) and as NACT ( ≤ 29y: 66.9%; 30–39y: 56.0%) in comparison with older patients regarding both overall CHT (60–69y: 37.5%; ≥ 70y: 17.6%) and NACT (60–69a: 25.5%; ≥ 70y 22.8%). Between 2008 and 2017, the proportion of patients in all age groups who had received NACT rose (Fig. 2), whereas CHT use declined overall, primarily in the age group between 40 and 70 years (Fig. 3).

### Response to neoadjuvant chemotherapy

**Fig. 4:**
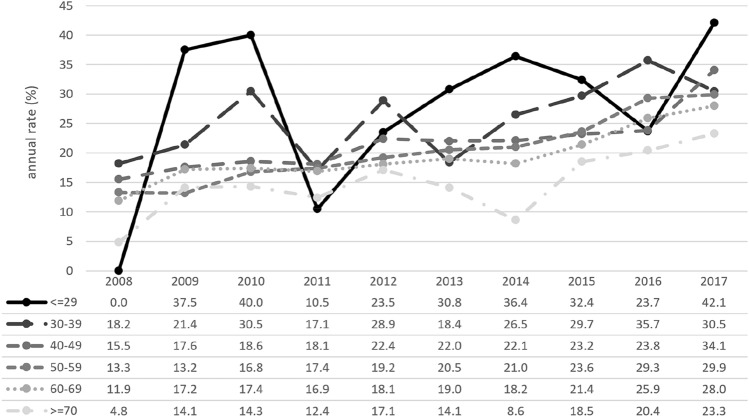
Rates for pathological complete response (pCR: ypT0 ypN0) after neoadjuvant chemotherapy, divided into six age groups (Group 1: ≤ 29 years; Group 2: 30–39 years; Group 3: 40–49 years; Group 4: 50–59 years; Group 5: 60–69 years; Group 6: ≥ 70 years; total *n* = 14,783)

Between 2008 to 2017, the rate of pCR (ypT0 ypN0) rose for all patients after NACT (*n* = 14,783) in all age groups. Overall, pCR rates were higher in younger patients than in older patients (Fig. 4). Across all ages, pCR rates were highest among patients with the tumor subtype HR– HER2+ , which affected 45.1% of patients compared with 34.0% and 30.4% of patients with the tumor subtypes HR– HER2– and HR+ HER2+ , respectively. Divided by age group, pCR rates sank with rising age (≤ 29y: 28.4% vs. ≥ 70y: 16.9%) (Table 2).

**Table 2:** Percentage of patients who achieved pathological complete remission (pCR, defined as ypT0 ypN0) after having received neoadjuvant chemotherapy (NACT), divided by age group and tumor subtype

Percentage of patients who achieved pCR after having received NACT, divided by age group and subtype					
	HR+ HER2−	HR+ HER2+	HR− HER2+	HR− HER2−	All subtypes
≤ 29 y	14.1	26.2	36.4	39.1	**28.4**
30–39 y	13.7	32.2	41.0	34.3	**27.6**
40–49 y	10.9	27.2	37.2	31.3	**22.5**
50–59 y	9.0	26.2	43.2	28.4	**22.6**
60–69 y	8.1	29.4	37.9	27.7	**21.3**
≥ 70 y	5.1	22.0	35.5	19.3	**16.9**
All ages	**10.8**	**30.4**	**45.1**	**34.0**	**30.8**

### Multivariable model

A multivariable logistic regression of factors that influence pCR achievement after NACT was performed (Table 3). Young age was found to be positively correlated with pCR; however, this finding was not statistically significant. The odds of achieving pCR significantly increased for patients with HER2+ and TN EBC compared with for patients with the HR+ HER2– subtype. Regarding the influence of caseload, a higher caseload was associated with lower odds of achieving pCR. These findings were statistically significant.

**Table 3:** Multivariable logistic regression, revealing factors that influence the achievement of pathological complete remission (vs. no pathological complete remission) (*n* = 8943)

	Odds ratio (95% CI)	*P* value
Age		
≤ 29 y	Reference	Reference
30–39 y	1.4123 (0.6372–3.0996)	0.3951
40–49 y	1.2531 (0.5812–2.6725)	0.5649
50–59 y	1.0184 (0.4728–2.168)	0.9629
60–69 y	0.8238 (0.3776–1.775)	0.6258
≥ 70 y	0.776 (0.3408–1.7477)	0.5452
Grading		
G1	Reference	Reference
G2	1.2277 (0.6229–2.4658)	0.5611
G3	1.2791 (0.6524–2.5516)	0.4818
Subtype		
HR+ HER2–	Reference	Reference
HR+ HER2+	1.8646 (1.3965–2.4914)	< 0.001
HR– HER2+	2.4872 (1.7914–3.4574)	< 0.001
HR– HER2–	1.6639 (1.2753–2.1714)	< 0.001
Hospital type		
University	Reference	Reference
Teaching hospital	1.1392 (0.8116–1.5995)	0.4514
Other	1.2352 (0.868–1.758)	0.2407
Annual caseload		
≤ 100 cases	Reference	Reference
101–250 cases	0.6035 (0.4275–0.855)	0.0043
> 250 cases	0.4867 (0.3418–0.6942)	< 0.001
Karnofsky index		
50	Reference	Reference
60	1.3289 (0.1142–15.5768)	0.8235
70	2.1119 (0.2917–16.3266)	0.4757
80	1.4683 (0.2377–9.8417)	0.6938
90	2.2939 (0.3789–15.1321)	0.3906
100	1.6069 (0.2662–10.5641)	0.6233

## Discussion

This study analyzed the impact of age on systemic treatment patterns for EBC using data from a large patient cohort in clinical routine in Germany.

Since the emergence of molecular classification systems [13], it has become evident that systemic therapy for EBC must be tailored according to individual risk factors, such as tumor stage and subtype. Gene-expression profiles have been implemented in clinical routine for cases for which no other criteria enable adequate adjuvant treatment with chemotherapy. Nonetheless, age remains an important factor in the complex process of decision-making for adjuvant and neoadjuvant systemic therapy treatment in EBC [14]. While most patients who are affected with EBC are between 40 and 70 years old, patients outside of this range — that is, both very young and elderly patients — might be at risk of overtreatment or undertreatment, both of which are associated with deviations from guideline-adherent treatment. To address specific challenges for these subgroups, recommendations have been established for elderly patients [15] and for younger patients [7, 16]. Age groups differ not only in their clinico-pathological characteristics, but also in demographic factors, such as life expectancy, time of diagnosis, and differences in individual screening and treatment patterns [17], as demonstrated by our patient characteristics (Table 1). Moreover, studies have shown that molecular subtypes have different distributions and prognostic effects in elderly EBC patients compared with in younger patients, and biomarkers therefore have different implications in elderly patients compared with in their younger counterparts [18]. Comparable to these finding, our data also revealed differences in the prevalence of tumor subtypes between age cohorts, with a higher rate of unfavorable subtypes (HER2+ and TN) having been found in younger patient cohorts (Table 1).

One study from Germany demonstrated that only about 3 out of 4 patients with EBC undergo guideline-adherent therapy, which results in unfavorable outcome parameters for patients with guideline violations [19]. A major subgroup with guideline violations seems to be patients with higher age [20–23]. Several comparable results have demonstrated that higher age remains a barrier to receiving chemotherapy for EBC, as has been shown, for example, in France [24], Denmark [25], Spain [26], and the US [27]. In Germany, the most important reason for discouraging patients from undergoing chemotherapy is somatic comorbidities and age > 75 years [19]. In general, relevant comorbidity prevalence upon EBC diagnosis increases with age and likely negatively influences the chances of receiving guideline-adherent systemic treatment [28].

Regarding outcomes, adjuvant chemotherapy in elderly patients is postulated to be beneficial, as has been shown for low-risk subgroups [29] and for patients with unfavorable tumor characteristics [30]. Upon examining outcome perspectives for extremely old EBC patients, these age groups also seem to profit from adjuvant chemotherapy, as results for patients > 75 years in South Korea [31] and for patients > 80 years in Singapore [32] have demonstrated. A recent analysis from the US revealed that chemotherapy is also associated with improved overall survival in node-positive, estrogen-receptor-positive elderly patients with multiple comorbidities [33]. In this context, higher recurrence rates in elderly patients compared with in younger post-menopausal women were explained by the under-use of systemic treatment in these groups [23].

When treating elderly patients with chemotherapy, the risk of hematotoxicity must be considered, specifically when using anthracyclines [34]. However, other risks seem to increase in elderly EBC patients who undergo chemotherapy, including acute kidney injury [35] and secondary haemato-oncological diseases [36]. Cardiotoxicity might be an additional problem for the application of trastuzumab in combination with standard chemotherapy, especially in HER2+ patients. Thus, in one US study, the highest rates of non-standard chemotherapy regimens in EBC were found among elderly women and were associated with fewer toxicity-related hospitalizations but with worse survival rates [37]. In contrast, the chemotherapy regimens used in women with EBC aged 70 and above in Germany appear to be relatively standardized and correspond to the recommendations given in the respective guidelines [38]. Survey results from outside Germany reveal a relevant lack of knowledge concerning the specific management of elderly patients affected by EBC [39].

Regarding pCR rates, age has an unfavorable impact on the chances of pCR, but acceptable rates are still possible, especially in HER2+ elderly patients [40]. These results are in line with our data, which reveal a general negative likelihood of pCR among patients with higher ages (Table 3) but no relevant decrease in pCR rates for patients with HER2+ tumors— in contrast to patients with HER2−  subtypes in higher age cohorts (Table 2). In the multivariable model, the trend of having lower chances of pCR among elderly patients is mainly driven by the lower prevalence of these HER2+ subtypes rather than by the elderly population itself (Table 3). The negative effect of age on pCR can, thus, be concluded to have most likely been factored out due to the increased occurrence of HR+ HER− with increasing age.

Therefore, when assessing the risks and benefits of chemotherapy for older patients, treatment must be adapted to general health and tumor biology rather than to age. In these cases, a professional geriatric assessment has been shown to benefit from therapy management [41]. It seems to be beneficial to evaluate individual risk factors in elderly EBC patients in order to avoid short-term mortality after adjuvant chemotherapy [42]. While undertreatment among elderly patients is often reported for systemic therapy, the opposite trend appears in surgical procedures, with continued overtreatment (e.g., in axillary management) causing unnecessary morbidity without any oncological benefit [43]. Moreover, for radiotherapy, this trend of reducing the therapy intensity is important: As one study demonstrated for patients aged 70 and older in low-risk EBC situations, breast irradiation after breast-conserving surgery can be avoided with a less-than-3% chance of local recurrence [44].

Younger women have poorer survival rates after breast cancer than older women: Previous research has demonstrated that young age is an independent risk factor for disease recurrence and death, although recent data suggest that this finding may not be true for all EBC subtypes [45] and that younger patients have higher proportions of HER2+ and TN subtypes than older women and are also more likely to be primarily diagnosed with advanced disease [46]. These findings are congruent with tumor characteristics in our patient cohorts (Table 1). In the literature, younger patients face higher rates of mastectomy and the use of chemotherapy, which indicates that more aggressive therapy is recommended or chosen for women in this age group in general [47]. Additionally, in these cohorts, EBC is more likely to have a hereditary background that might influence the decision to undergo treatment with a more aggressive approach [48].

Future clinical trials that focus on these specific subgroups appear to be necessary in order to find proper treatment strategies. Some prospective trials have already been established, such as the UK-based POSH study, which addresses younger patients in high-risk situations [49].

Our study has several limitations: Although the German registry is very large and covers the entire country, it is still only a sample and is not a comprehensive mandatory registry. Therefore, the results may not be entirely representative of all institutions [50]. Unfortunately, as we have a benchmarking database, information on individual patient status (e.g., comorbidities) and clinical tumor stage is not available. Thus, we were not able to adjust our data by considering these baseline patient characteristics.

## Conclusion

The results of this large, nationwide cohort reveal both a relevant discrepancy concerning the use of chemotherapy based on age and the risk of undertreatment or overtreatment among the subgroups of very young patients and elderly patients with an EBC diagnosis.

## Data Availability

The data that support the findings of this study are available from the corresponding author upon reasonable request.
